# MUC2 protein expression status is useful in assessing the effects of hyperthermic intraperitoneal chemotherapy for peritoneal dissemination of colon cancer

**DOI:** 10.3892/ijo.2012.1334

**Published:** 2012-01-16

**Authors:** YUKA FUJISHIMA, TAKANORI GOI, YOUHEI KIMURA, YASUO HIRONO, KANJI KATAYAMA, AKIO YAMAGUCHI

**Affiliations:** First Department of Surgery, University of Fukui, 23-3 Eiheiji-cho, Yoshida-gun, Fukui, Japan

**Keywords:** colon cancer, mucin, hyperthermic intraperitoneal chemotherapy, peritoneal dissemination

## Abstract

We conducted a molecular biological investigation to determine the outcomes of hyperthermic intraperitoneal chemotherapy (HIPEC) treatment, and whether it is effective in all cases for patients with peritoneal dissemination of colon cancer. In the HIPEC group, the 3-year survival rate was 39.2%, whereas in the non-HIPEC group the 3-year survival rate was 15.6%. MUC2 expression was investigated in the HIPEC group, in patients positive for MUC2 expression, and the 3-year survival rate was 0.0%, while in patients negative for MUC2 expression, the 3-year survival rate was 61.1%. In addition, as a result of introducing MUC2-siRNA into a colon cancer cell line with high expression of the MUC2 gene, the cell death rate from heat and anticancer agents increased 40% in comparison with colon cancer cells in which scrambled siRNA had not been introduced. HIPEC therapy is thought to be effective in prolonging survival in patients with peritoneal dissemination of colon cancer, and MUC2 expression is thought to be useful as an indicator to assess its effectiveness in colon cancer cells.

## Introduction

The prevalence and mortality of colon cancer are the highest among malignant tumors in Western countries and Japan (1). Currently, resection of metastatic foci and chemotherapy have been shown to be effective in the treatment of liver and lung metastases and other hematogenous metastases (2). In colon cancer patients, however, the incidence of peritoneal dissemination is 7% of initial colon cancers and 4–19% of recurrent colon cancers, but no effective treatment modalities for this peritoneal dissemination have been established, which is a major problem (3,4). At present, cytoreduction and hyperthermic intraperitoneal chemotherapy (HIPEC) are conducted for peritoneal dissemination, and there are studies of improved outcomes and long-term survival in some patients (5,6). Sugarbaker scored peritoneal dissemination anatomically and with consideration of tumor diameter, and investigated the contribution to outcome when cytoreduction and HIPEC were conducted (7). Recently, Terence *et al* scored clinical symptoms, extent of carcinomatosis, and tumor pathology in patients who underwent HIPEC, and reported that the effect differs depending on the score (8). As there have been no reported molecular biological investigations on the efficacy of HIPEC, we decided to search for useful molecules.

Mucins have attracted attention as substances that play a large role in the protective mechanisms of normal colonic mucosa. Mucins are classified according to basic core protein type. This core protein is abbreviated MUC, and 21 types of mucin have been reported to date. Mucins are broadly classified into two types: 1) Secretory mucins, which are secreted from epithelial cells and are a main component of mucus in the traditional sense, and these mucin molecules form gels; and 2) Membrane-bound mucins, which bind to cell membranes. Mucin molecules have an extracellular domain, a transmembrane domain and an intracellular domain, and exist in a form that passes through the cell membrane (9,10).

Among these, the secretory type MUC2 is a major mucin that is recognized to be expressed in the normal intestinal tract, but expression of MUC5AC and MUC3 is also seen. The mucosal layer of organs that contact the external world is protected physically by mucins, and they are thought to be important molecules in biological defense (11,12). Mucins are also reported to be involved in carcinogenesis of the intestinal tract, one of the causes is thought to be that the intestinal mucosa is susceptible to chronic inflammation when mucins are deficient (13,14). Thus, mucins secreted in normal mucosa are thought to act to protect the body’s own cells from external influences. Moreover, the expression of mucin family proteins is not uniform in various malignant tumors, and expression of MUC proteins is conjectured to serve certain function in cancer cells (15,16). Thus, by enhancing expression in cancer cells themselves, they are also thought to protect against assault from anticancer agents or other substances from the external world, contributing to anticancer agent resistance and worsening prognosis. In the present study, therefore, we investigated the effectiveness of HIPEC and whether expression of mucin family proteins are involved.

## Patients and methods

### Patients

The subjects were 935 colon cancer patients who had undergone resection for colorectal cancer at the First Department of Surgery, University of Fukui, Japan, between 1994 and 2010 (Table I). Surgical specimens of the peritoneal dissemination were obtained from 37 patients with peritoneal metastases as the only distant metastasis of primary colon cancer. HIPEC was performed in 22 patients with peritoneal dissemination as the only synchronous distant metastasis in cases of primary colon cancer at our hospital from 1994 to 2010. The outcome was then compared with that of patients (15 cases) who did not receive HIPEC. Cancers were reviewed and graded by two pathologists using criteria recommended by the general rules of clinical and pathological studies on cancer of colon, rectum and anus for histological type, lymphatic invasion and venous invasion (17). The research was performed in accordance with the humane and ethical rules for human experimentation that are stated in the Declaration of Helsinki.

### HIPEC procedure

This procedure allowed the abdominal cavity to be extended widely enough to allow perfusate to spread throughout the peritoneal cavity (18). Two liters of saline contained 150 mg of cisplatin, 20 mg mitomycin C, and 200 mg of etoposide. An additional 2 litres of the same infusate were heated in a waterbath and pumped into circulation between the abdomen and a reservoir at ~500 ml per minute. The temperature at several points of the peritoneal surface was maintained at appromimately 43°C by controlling the temperature of the water bath and the speed of the pump. Abdominal temperatures were measured at the serosal surface in the subphrenic space and the cavity of Douglas, and the temperature of the infusate was also measured in the inflow tube, outflow tube, and water bath. The thermal dose (TD) obtained during the treatment was calculated simultaneously during HIPEC and expressed in terms of equivalent time at 43°C (19). HIPEC was performed until the TD reached 40 min.

### Immunohistostaining

The patients with peritoneal dissemination as the only synchronous distant metastasis in cases of primary colon cancer were analyzed for MUC1, 2, 3, 4, and 5AC protein expression. Surgical specimens of the peritoneal dissemination prepared from formalin-fixed, paraffin-embedded tissues were analyzed for protein expression by the streptavidin-biotin peroxidase method (20). The expression was interpreted as positive when the protein was expressed in >30% of the cancer cells.

### Antibody

The following antibodies were used: anti-human MUC1, 2, and MUC5 (Novocastra, UK), MUC3 (Santa Cruz Biotechnology, CA, USA), MUC4 (Invitrogen, CA, USA).

### Cell culture

The human colon cancer cell lines (SW620, colo205, and LoVo) were cultured at 37°C in 5% CO_2_ in RPMI-1640 or DMEM medium containing 10% fetal bovine serum.

### Total RNA extraction, RT-PCR analysis

Total RNA was extracted from cells using Isogen (Wako, Japan) (21). Single strand cDNA prepared from 3 μg of total RNA using Moloney murine leukemia virus reverse transcriptase (Gibco-BRL, MD, USA) with an oligo (dT) primer-14 was used as the template for the polymerase chain reaction (PCR). The primers for PCR to amplify MUC2 gene-coding regions were: The 5′ primer, MUC2-AX: TGCCTGGCCCTGTCTTTG and the 3′ primer, MUC2-BX: CAGCTCCAGCATGAGTGC. Thirty cycles of denaturation (94°C, 1 min), annealing (50°C, 0.75 min), and extension (72°C, 2 min) were carried out in a thermal cycler (PTC-100, programmable thermal controller, NJ Research Inc., MA, USA). GAPDH amplification was used as internal PCR control with 5′-GGGGAGCCAAAAGGGTCATCATCT-3′ as the sense primer and 5′-GACGCCTGCTTCACCACCTTCTTG-3′ as the antisense primer. Thirty cycles of denaturation (94°C, 1 min), annealing (50°C, 1.5 min), and extension (72°C, 2 min) were carried out in a thermal cycler. PCR product (10 μl) was resolved by electrophoresis in 12% acrylamide gel. The sequencing was performed on PCR products that showed the bands in RT-PCR analysis. Sequence analysis showed the presence of the MUC2 gene.

### Transfection

The cells were cultured with RPMI-1640, 10% FBS and 1X penicillin/streptomycin at 37°C and 5% CO_2_ incubator. MUC2-siRNA (Invitrogen) and scrambled (SCR)-siRNA (Invitrogen) were purchased. Cells were transfected with 100 nM of MUC2-siRNA, or SCR-siRNA with Lipofectamine 2000 reagent (Invitrogen) in accordance with the manufacturer’s protocol. SCR-siRNA was used as negative control.

### Heat and anticancer agent treatment

The cells were plated onto a 96-well plate at 1×10^4^ and incubated for 12 h. The cells were treated with cisplatin of 100 μg/ml at 43°C (5% CO_2_ incubator) for 24 h.

### MTS analysis

To assess cell proliferation, Cell Titer 96 Aqueous Non-radioactive Cell Proliferation Assay (Promega, Germany) was used according to the manufacturer’s instructions. MTS solution (20 μl) was added to each well (96-well plate) and the plates were incubated at 37°C for 1.5 h. The absorbance of the product formazan, which is considered to be directly proportional to the number of living cells in the culture, was measured at 490 nm using a Microplate Reader (Molecular Devices, CA, USA).

### Statistical considerations

Survival time was calculated using the Kaplan-Meier method, and log-rank test was used to compare the curves of the survival times.

Other characteristics of the two treatment arms were compared using the chi-square test. Values of P<0.05 were considered as statistically significant.

## Results

### Relationship between survival rate and whether HIPEC was used in the patients with peritoneal dissemination of colon cancer

**Figure 1:**
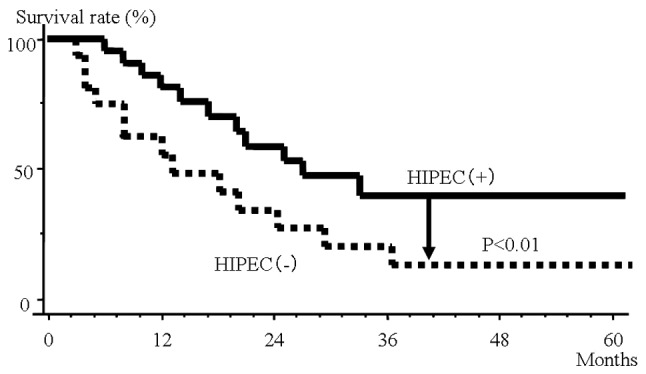
Overall the survival curves of patients with HIPEC therapy. The 3-year survival rate was 15.6% in the non-HIPEC group, whereas the 3-year survival rate was 39.2% in the HIPEC group.

Fig. 1 shows the survival rates in the HIPEC and non-HIPEC groups among all subjects with peritoneal dissemination. In the non-HIPEC group, the median survival time was 13 months and the 3-year survival rate was 15.6%, whereas in the HIPEC group the median survival time was 24 months and the 3-year survival rate was 39.2%. The HIPEC group thus had significantly better outcomes.

### Investigation of expression of mucin proteins in dissemination foci of patients with peritoneal dissemination of colon cancer

**Figure 2:**
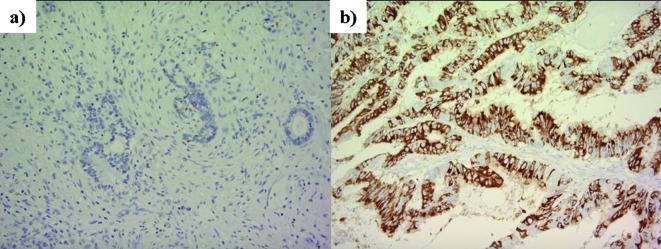
Immunostaining of MUC2 isoform in colon cancers. (a) MUC2 isoform was not expressed in cancer cells. (b) The expression of MUC2 was expressed in the cytoplasm and cell membrane of cancer cells.

**Figure 3:**
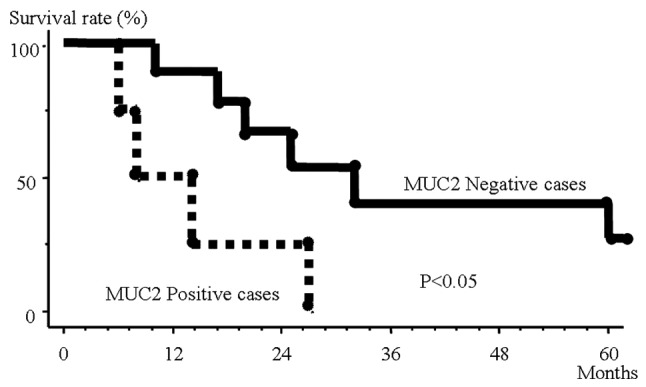
Overall survival curves of patients treated with HIPEC therapy subdivided according to expression of MUC 2. The patients with MUC2-positive tumors had poorer prognosis than those with MUC2-negaitive counterparts.

Fig. 2 shows images of positive and negative immunohistochemical staining using anti-MUC2 antibody in dissemination foci in patients with peritoneal dissemination of colorectal cancer, and Fig. 3 shows their incidence. Of the 22 patients, MUC1 protein expression was seen in 19 patients (86.4%), MUC2 in 10 (45.5%), MUC3 in 10 (45.5%), MUC4 in 15 (68.2%), and MUC5AC was seen in 15 (68.2%).

### Investigation of MUC2 protein expression and outcome in colon cancer patients with peritoneal dissemination who underwent HIPEC

**Figure 4:**
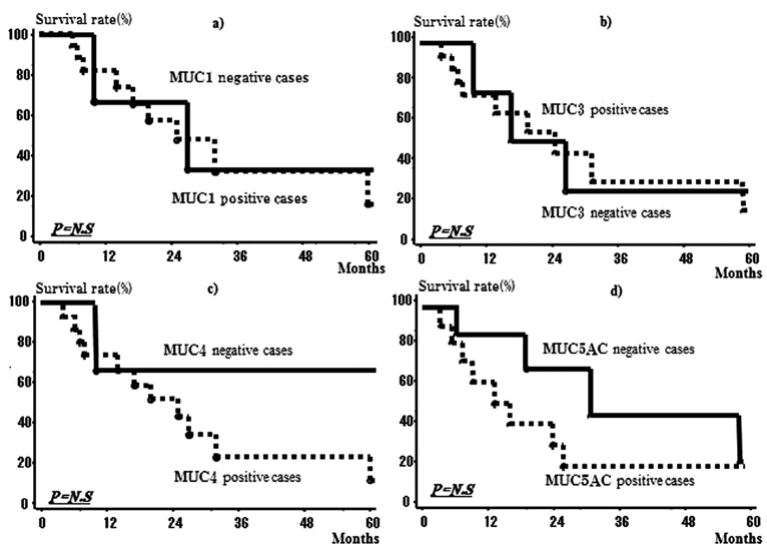
Overall survival curves of patients treated with HIPEC therapy subdivided according to expression of MUC1, 3, 4, and 5AC. There was no significant correlation between MUC1 (a), 3 (b), 4 (c), and 5AC (d) immunoreactivity and prognosis.

Expression of MUC2 protein and outcome was investigated in the patients with peritoneal dissemination of colon cancer in the HIPEC group. In patients positive for MUC2 expression, the MST was 14 months and the 3-year survival rate was 0.0%, whereas in patients negative for MUC2 expression, the 3-year survival rate was 61.1% (Fig. 3). Patients negative for MUC2 expression thus had a significantly better outcome. In the non-HIPEC group, no relationship was seen between outcome and expression of MUC2 protein. There was no significant difference between survival time and presence or absence of MUC1, 3, 4 and 5AC expression (Fig. 4a–d).

### Investigation of MUC2 mRNA expression in colon cancer cell lines

**Figure 5:**
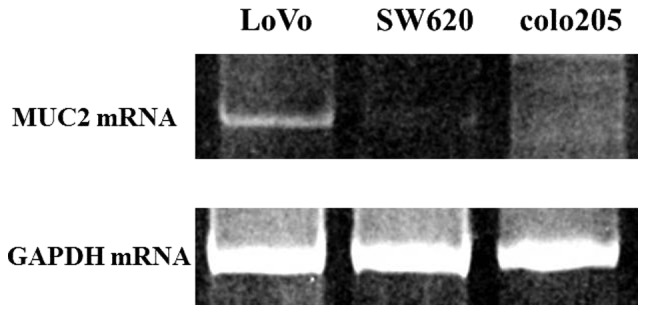
MUC2 mRNA expression in colon cancer cell lines. MUC2 mRNA expression was observed in the colon cancer cell lines: LoVo. No expression was seen in SW620 and colo205.

The results of an investigation of MUC2 mRNA expression in three different colon cancer cell lines are shown in Fig. 5. The highest expression of MUC2 was seen in the LoVo cell line. No expression was observed in SW620 and colo205.

### Effects of MUC2-SiRNA introduction on heat and anticancer agent

**Figure 6:**
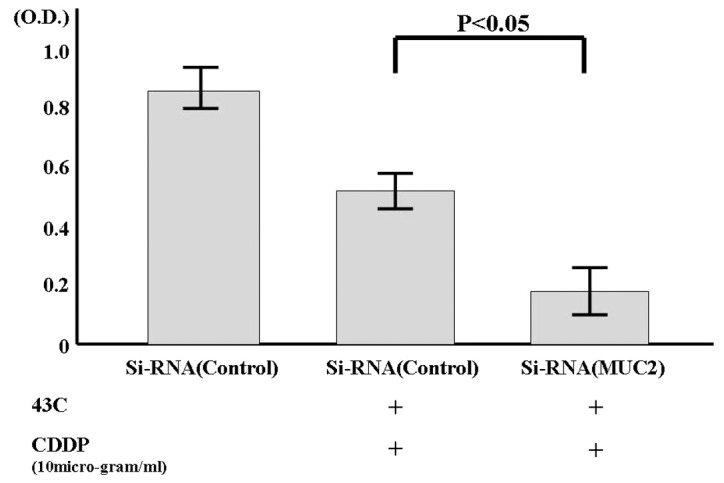
The living cells on heat and anticancer agents. The percentage of living cells among the colon cancer cells with scrambled SiRNA (cultured at 37°C) was taken to be 100%. The percentage in the cells with scrambled SiRNA was 63% (0.53 O.D.). The percentage in the cell with SiRNA-MUC2 was 20% (0.17 O.D.).

When SiRNA-MUC2 was introduced into the LoVo cell line, which showed high MUC2 expression, the expression of MUC2 mRNA decreased as shown in Fig. 6. The percentage of living cells was investigated after culturing these cells for 1 day at 43°C in the presence of an anticancer agent. When the percentage of living cells among the cells with scrambled SiRNA (cultured at 37°C) was taken to be 100% (0.84O.D.), the percentage in the cells with scrambled SiRNA was 63% (0.53O.D.), and that in the cells with MUC2-SiRNA was 20% (0.17O.D.).

## Discussion

In recent years, with advances in anticancer agents and molecularly targeted drugs for chemotherapy, improvements have been seen in outcomes for unresectable colon cancer, particularly hematogenous metastasis (2). However, there are no reported large-scale trials showing clear improvements in outcome for peritoneal dissemination, and there is no established treatment. Cytoreduction and HIPEC are now conducted in these patients, and reports of their efficacy are occasionally seen (5,6). At our hospital, HIPEC has been performed for colon cancer patients with peritoneal metastasis as the only distant metastasis, and a significant effect has been seen when compared with patients who did not receive HIPEC (5).

HIPEC also has mixed efficacy, being effective in some cases and ineffective in others. If it were possible to judge the cases in which it would be effective, it could reasonably be considered to act in extending the lives of patients. Therefore, the efficacy of HIPEC was investigated from a molecular biological perspective, considering the importance of the properties of the cancer cells themselves; that is, their gene/protein expression state. We focused on mucin family proteins in healthy cell membranes, which are thought to protect the cell from insults from the outside. MUC1, 2, 3, 4 and 5A are thought to have a particularly close relationship with colon tissue (12,22–24), and from investigation of these proteins, it is thought that MUC2 expression is important in the effectiveness of HIPEC therapy.

MUC2 is a secretion type mucin, and in normal mucosa, it is thought to cover the surface of soft mucosa and provide a physically barrier, protecting the organism by constantly washing off the mucosal surface (11). Thus, it may be that MUC2 proteins, by being secreted on the surface of colon cancer cells, protect the cancer cells at least partially from the effects of chemotherapy, which is thought to be related to the effects of HIPEC in this study.

We investigated the effects of HIPEC when RNAi was used to block the activity of the MUC2 gene. When MUC2 gene expression was inhibited, HIPEC was demonstrated experimentally to have increased effectiveness, and the protection of MUC2 covering the surface of cancer cells decreased. The degree to which cells were affected by HIPEC was thought to have increased.

In the present study, the effectiveness of HIPEC was seen to decrease when MUC2 protein expression was seen in colon cancer cells and, conversely, to increase when MUC2 protein expression was not seen. Thus, MUC2 expression may be useful as an indicator in determining whether HIPEC is indicated.

## Figures and Tables

**Table I:** Patient characteristics.

	HIPEC cases	Non-HIPEC cases
No. of patients	22	15
Gender (M/F)	10/22	8/7
Age (years)	54.1 (31–74)	70.5 (47–81)
Differentiation		
Well, mod	17	6
Others	5	9
Synchronous/metachronous	17/5	15/0
